# Associations between self-referral and health behavior responses to genetic risk information

**DOI:** 10.1186/s13073-014-0124-0

**Published:** 2015-01-31

**Authors:** Kurt D Christensen, J Scott Roberts, Brian J Zikmund-Fisher, Sharon LR Kardia, Colleen M McBride, Erin Linnenbringer, Robert C Green, for the REVEAL Study Group

**Affiliations:** Division of Genetics, Brigham and Women’s Hospital and Harvard Medical School, EC Alumnae Building, Suite 301, 41 Avenue Louis Pasteur, Boston, MA 02115 USA; Department of Health Behavior and Health Education, University of Michigan School of Public Health, Ann Arbor, MI 48109 USA; Department of Epidemiology, University of Michigan School of Public Health, Ann Arbor, MI 48109 USA; Department of Behavioral Sciences and Health Education, Rollins School of Public Health, Atlanta, GA 30322 USA; Division of Public Health Sciences, Department of Surgery, Washington University School of Medicine, St Louis, MO 63110 USA; Division of Genetics, Brigham and Women’s Hospital/Harvard Medical School/Partners Personalized Medicine, Boston, MA 02115 USA

## Abstract

**Background:**

Studies examining whether genetic risk information about common, complex diseases can motivate individuals to improve health behaviors and advance planning have shown mixed results. Examining the influence of different study recruitment strategies may help reconcile inconsistencies.

**Methods:**

Secondary analyses were conducted on data from the REVEAL study, a series of randomized clinical trials examining the impact of genetic susceptibility testing for Alzheimer’s disease (AD). We tested whether self-referred participants (SRPs) were more likely than actively recruited participants (ARPs) to report health behavior and advance planning changes after AD risk and *APOE* genotype disclosure.

**Results:**

Of 795 participants with known recruitment status, 546 (69%) were self-referred and 249 (31%) had been actively recruited. SRPs were younger, less likely to identify as African American, had higher household incomes, and were more attentive to AD than ARPs (all *P* < 0.01). They also dropped out of the study before genetic risk disclosure less frequently (26% versus 41%, *P* < 0.001). Cohorts did not differ in their likelihood of reporting a change to at least one health behavior 6 weeks and 12 months after genetic risk disclosure, nor in intentions to change at least one behavior in the future. However, interaction effects were observed where ε4-positive SRPs were more likely than ε4-negative SRPs to report changes specifically to mental activities (38% vs 19%, p < 0.001) and diets (21% vs 12%, p = 0.016) six weeks post-disclosure, whereas differences between ε4-positive and ε4-negative ARPs were not evident for mental activities (15% vs 21%, p = 0.413) or diets (8% versus 16%, *P* = 0.190). Similarly, ε4-positive participants were more likely than ε4-negative participants to report intentions to change long-term care insurance among SRPs (20% vs 5%, p < 0.001), but not ARPs (5% versus 9%, *P* = 0.365).

**Conclusions:**

Individuals who proactively seek AD genetic risk assessment are more likely to undergo testing and use results to inform behavior changes than those who respond to genetic testing offers. These results demonstrate how the behavioral impact of genetic risk information may vary according to the models by which services are provided, and suggest that how participants are recruited into translational genomics research can influence findings.

**Trial registration:**

ClinicalTrials.gov NCT00089882 and NCT00462917

**Electronic supplementary material:**

The online version of this article (doi:10.1186/s13073-014-0124-0) contains supplementary material, which is available to authorized users.

## Background

Genetic testing is increasingly accessible in both clinical settings and the consumer marketplace, and hopes persist that disclosure of genetic predispositions for disease will empower individuals to change behaviors to reduce their disease risks. While a number of studies have suggested that there is little in the way of significant health behavior change following genetic testing for common diseases [1,2], a notable exception has been genetic susceptibility testing for Alzheimer’s disease (AD). Multiple separate trials conducted as part of the Risk Evaluation and Education for Alzheimer’s Disease (REVEAL) study have shown that cognitively normal adults with an affected parent or sibling who learned of an increased genetic risk for AD are more likely than those at population risk or those receiving non-genetic risk assessments to report changes in putative AD-prevention behaviors [3,4]. Furthermore, the study showed that individuals who learn of an increased genetic risk are more likely to report changes to advance planning [5,6]. These findings have led commentators to cite AD genetic susceptibility testing as an example of the personal utility genetic risk assessments can provide [7-9].

The inconsistencies between REVEAL study findings and research from the field at-large raises questions about what may differ between studies. Certainly, the lack of well-proven prevention and treatment strategies distinguishes AD from other common diseases, but the public tends to believe that lifestyle, diet, and mental activity are important determinants of AD risk [10]. It is also possible that the REVEAL study enrolled participants who were more motivated to pursue lifestyle modifications than other studies. While nearly all studies of genetic risk disclosure have enrolled self-selected populations [1], the REVEAL study is distinctive in its proportion of participants who self-referred to the study compared to the proportion who were actively recruited. Individuals who self-refer to an intervention not only tend to have stronger personal and family histories of disease [11-14] and stronger concerns about developing the disease [11], but are also more likely to engage in intervention activities [15,16]. These factors may help explain why self-referred populations are frequently more likely to report behavior changes following an intervention than actively recruited populations [12,15].

Understanding how individuals who proactively seek a genetic risk assessment may differ from individuals who are responding to investigator-driven offers of testing may help reconcile inconsistent findings across studies and inform future models for genetic services. The analysis that follows compared self-referred participants (SRPs) and actively recruited participants (ARPs) from the second and third trials of the REVEAL study. First, we examined the characteristics of each enrolled cohort, expanding upon analyses of data from the first REVEAL study trial – which enrolled ARPs exclusively from research registries – by comparing a wider range of demographic and psychosocial characteristics. We then compared the percentage of participants who received a genetic risk assessment. Finally, we examined associations between SRPs and ARPs in terms of their behavioral outcomes, namely changes to mental activities, diet, exercise, dietary supplement use, medications, long-term care insurance coverage, and retirement plans. We hypothesized that self-referred participants would be more likely to report changes to health behaviors and advance planning than ARPs.

## Methods

### Overview and study population

We conducted secondary analyses on data from the REVEAL study, a series of multicenter randomized controlled trials examining the psychological and behavioral impact of providing AD genetic risk information based on *APOE* genotyping [17-19]. Analyses were conducted on data from the second and third study trials (data from the first trial were omitted because the scales and timing used to measure health behavior changes and beliefs about AD and genetic testing greatly differed from those used in the second and third trials). In each trial, study sites recruited participants through a combination of strategies described in more detail below. Individuals with two or more AD-affected first degree relatives (FDRs), from families where the average AD onset age was under 60 years, or who scored below an education-adjusted 87 on the Modified Mini-Mental State Examination [20] were excluded from participation. Individuals with severe psychiatric disorders, defined as scoring above 25 on the Beck Anxiety Inventory [21] or above 26 on the Center for Epidemiological Studies-Depression Scale [22], were also excluded. In the second trial, which tested condensed education and disclosure protocols, eligibility criteria required all participants to have a single AD-affected FDR. In the third trial, which compared phone versus in-person disclosure as well as disclosing pleiotropic versus AD-only information, approximately 25% of participants had no AD-affected FDRs, while 75% had one AD-affected FDR. A more comprehensive overview of the REVEAL study has been published previously [17].

### Study design

The multidisciplinary REVEAL study group designed the study protocol and risk disclosure procedures, which was reviewed by an external advisory board. The Boston University Medical Campus Institutional Review Board (IRB), Howard University Medical IRB, University Hospitals IRB, University of Michigan Medical School IRB, and Weill Cornell Medical College IRB approved the final protocol, Participants provided informed consent by telephone at the time of study enrollment, then again in writing prior to the blood draw for genotyping.

After completion of a phone interview and written pre-education questionnaire, participants received education about testing. Participants received information (shown in Additional file 1) about (i) the definition of AD; (ii) general risk factors for AD and the general population’s level of risk; (iii) *APOE* genotype and its implications for risk of AD; (iv) procedures involved in *APOE* testing; (v) a preview of what would be provided in their risk assessment; and (vi) known benefits, risks, and limitations of *APOE* genotype testing, including the potential for companies and employers to ask for results and ‘deny insurance coverage or change your policy rates’ [23] (materials were not revised after the passage of the Genetic Information Nondiscrimination Act given ongoing concerns about disability and long-term care insurance [24]). Participants then met individually in person with a genetic counselor. Those still interested in pursuing a genetic risk assessment provided blood, which was genotyped at a Clinical Laboratory Improvement Amendments (CLIA)-certified facility. Approximately one month after the blood draw, participants received risk curves tailored to their gender, race, AD family history (whether or not the participant had an AD-affected FDR), and genotype [25,26]. They were also told their *APOE* genotype and numeric estimates of their cumulative lifetime (potential range: 6% to 73%) and remaining risk for AD (cumulative incidence from current age to the age of 85 years). Following genetic risk disclosure, REVEAL study researchers followed participants for the period of one year.

### Measures

#### Demographic characteristics

Participants self-reported all demographic characteristics, including age, race, gender, education, income, employment status, and number of AD-affected relatives. All information was collected at the point of enrollment except for education, income, and employment status, which were collected during the phone interview.

#### Recruitment cohort

At enrollment, study staff queried participants about how they ‘heard about the REVEAL study.’ The coordinator or assistant then categorized verbal responses at the time of enrollment into one of seven response categories: (a) ‘From another research study at this hospital,’ (b) ‘Someone from the study talked to me in the waiting room,’ (c) ‘Someone from the study called me at home or work,’ (d) ‘From a brochure or advertisement,’ (e) ‘Someone from the study gave a presentation,’ (f) ‘From a friend,’ or (g) ‘Other,’ with open-ended descriptions that were classified later (Table 1). Subjects were recoded as SRPs if the participant initiated the enrollment interaction (for example, called after reading about the study in the newspaper or on a website) or ARPs if REVEAL study personnel initiated the enrollment discussion (for example, contacted through a research registry, approached in neurology waiting room). Participants lacking data about recruitment were classified as ‘unknown’ and omitted from cohort comparisons.

**Table 1:** **Sources for the self-referred and actively recruited cohorts**

**Classification**	**Description**	**n**
**Self-referred participants (n = 546)**		
Brochure or advertisement	Participants saw recruitment brochures that study staff left in neurology clinic waiting rooms and related locations or responded to newspaper advertisements or flyers about the study	233
Media*	Participants read about the study in a newsletter, newspaper article or book	91
Web site*	Participants (a) found a website created at one of the participating study sites; (b) visited a news or entertainment website that was discussing the REVEAL study; (c) found study details on a website that helped studies enrol participants (for example, ClinicalTrials.gov); or (d) said they had learned about the study ‘online,’ ‘on the internet,’ or through a ‘Google search’ without being more specific	64
Study presentation	Participants attended a community event to raise awareness about AD where study personnel presented	49
Friend	Participants learned about the study from acquaintances who were already participating or heard about the study in the media	49
Acquaintance*	Participants mentioned a specific individual who told them about the REVEAL study	37
Health fair*	Participants approached a booth at a local health fair set up by Howard University	10
Wait list*	Boston University waitlisted individuals who had wanted to participate in the first REVEAL study trial	10
Self-referred*	Study personnel labeled participants as ‘self-referred’ without elaboration	3
**Actively recruited participants (n = 249)**		
Another research study	Researchers specializing in neurology and AD discussed the REVEAL study with patients enrolled in a separate study	149
Called at home or work	Study personnel called individuals who provided contact information for research purposes, usually through membership in a research registry	35
Provider referral*	A nurse, physician, or genetic counselor referred the participant to the study	25
Mailing*	Participants received mailings because they (a) had relatives who participated in the AD Anti-Inflammatory Prevention Trial (ADAPT), (b) were members of the Michigan AD Research Center registry, or (c) were members of the Michigan Dementia Coalition	15
Clinic intake*	Howard University personnel classified individuals they had approached at neurology clinics this way	12
Alzheimer’s Disease Center (ADC) referral*	The Boston University ADC referred individuals to the REVEAL study who (a) wanted to participate in another study that was already closed or for which they did not qualify, or (b) accompanied AD-affected relatives to ADC appointments for another study	7
Approached in the waiting room	REVEAL study personnel approached families in waiting areas of neurology and geriatrics clinics	6

*Classification was based on secondary coding of open-ended responses after an initial classification of ‘other.’

#### Beliefs about Alzheimer’s disease and genetic testing

During enrollment, participants verbally provided the following data.

- *Perceived susceptibility.* Participants responded to the question, ‘On a scale of 0 to 100%, what do you think your chance of developing AD during your lifetime is?’ in the second trial and ‘Out of 100 people just like you, how many of them do you think will develop AD in their lifetime?’ in the third. Analyses of perceived susceptibility are stratified by trial given these differences in wording.
- *Perceived seriousness.* Participants rated their agreement with the statement, ‘AD is the worst disease I can think of,’ on a five-point scale from strongly disagree (1) to strongly agree (5).
- *Alzheimer’s disease concern*. Participants rated their agreement to statements on a four-item scale (Cronbach α = 0.65) implemented in prior research on FDRs of AD patients [10]. Items included statements such as ‘I am concerned that I will develop AD’ and ‘I believe that I will someday develop AD.’ Higher scores signified greater AD concern.
- *Interest.* Study staff asked participants, ‘In general, do you think you would be interested in having a genetic test to assess your chance of developing AD?’ Responses were dichotomized as ‘yes’ versus ‘no’ and ‘maybe’ combined.

During the telephone interview, participants verbally provided the following data.

- *Alzheimer’s disease attentiveness.* Participants responded to the question, ‘Presently, how often do you think about getting AD?’ on a four-point scale from 1 (not at all/rarely) to 4 (a lot).
- *Coping self-efficacy.* On an open-ended question, participants rated their certainty that they would be able to cope with receiving a genetic test result that indicated an increased chance of developing AD from 0% (certain that they could not cope) to 100% (certain that they could cope).

Participants provided the following data on the pre-education questionnaire.

- *Perceived pros and cons.* Participants rated the importance of 11 reasons ‘why someone might take a genetic test for AD’ (pros) and 10 reasons ‘why someone might not want to take a genetic test for AD’ (cons) using scales developed from *BRCA* testing research and analyzed previously (Cronbach’s α = 0.83 for pros, 0.81 for cons) [27]. Scores on individual items ranged from 1 (not at all important) to 5 (extremely important), with means used in analyses.
- *Causal beliefs.* Participants rated the importance of ‘genetics/heredity’ or ‘lifestyle (for example, diet, exercise, smoking)’ for increasing one’s risk of AD. Scores ranged from 1 (not important) to 5 (very important).
- *Expectations.* The pre-education questionnaire asked, ‘What do you hope to get out of your risk assessment experience?’ Participants checked yes or no about expectations about receiving ‘reassurance’ and ‘help in decision making.’

### Behavioral responses

#### Health behaviors

On a written questionnaire administered 6 weeks after genetic risk disclosure, participants checked yes or no about whether they had made changes to preventive measures, including mental activities, diet, exercise, vitamin usage, herbal supplement usage, and medications. On the 12-month follow-up questionnaire, participants indicated whether they were continuing changes reported at 6 weeks, whether any new behavior changes had been made since the 6-week follow up, and whether they had plans to change behaviors in the future. Participants were classified as having changed a behavior at 12 months if they reported continuing a change reported at 6 weeks or if they reported making a new change between the 6-week follow-up and the 12-month follow-up. Data on vitamin and herbal supplement usage were combined and are reported as ‘dietary supplements’ based on taxonomy used in administration of the National Health and Nutrition Examination Survey (NHANES) [28].

#### Advance planning

Participants checked yes or no on the 12-month follow-up questionnaire about whether they had changed, or planned to change, long-term care insurance coverage or retirement plans related to the results of the genetic risk assessment.

#### Prevention discussions

Study clinicians noted whether ‘preventative measures’ were discussed with participants during results disclosure by checking the corresponding box on a chart note completed at the end of each disclosure session.

### Data analysis

Bivariate analyses using chi-squared tests, Wilcoxon rank-sum tests, linear regression, and logistic regression analyses were used to compare the demographic and psychosocial profiles of SRPs and ARPs at baseline. These techniques were also used for bivariate analyses that identified factors associated with study dropout before disclosure of genetic risk information. Logistic regression models, which controlled for demographic and psychosocial factors that varied by recruitment cohort as well as experimental variables (that is, randomization status), were used to examine changes to health behaviors and advance planning, including tests for interaction effects between cohort and genetic risk status. Logistic regression, controlling for the same demographic and experimental factors, was also used to test for differences in continuation rates of behavior changes reported at 6 weeks, and also to test for differences in likelihood of discussing preventive measures during the disclosure session. Associations between genotype and behavioral outcomes of interest were considered to differ by cohort only if *APOE*-cohort interactions were significant at α = 0.05. Analyses did not account for multiple hypothesis testing given that the studies were powered for testing primary hypotheses about psychological outcomes (that is, measures of anxiety and depression) rather than the logistic regression analyses presented here.

Per protocol analyses were conducted using R version 3.1.1 such that tests for health behavior change analyses included only participants who received genetic risk information [29]. We imputed item non-response using fully conditional specification using R package *mice* 2.22 [30], running 20 iterations to create each of 100 imputed datasets. Variables that were used to calculate joint probabilities for multiple imputation of missing data from the pre-education survey were selected using an inclusive strategy [31], and included recruitment cohort, study round, dropout stage, demographic factors, and psychosocial characteristics. Variables that were used to calculate joint probabilities for multiple imputation of missing behavioral data included all variables included in final logistic regression models, as well as study dropout stage.

## Results

### Cohort characteristics at enrollment

Across the two trials, 249 ARPs and 546 SRPs enrolled in the study, with sources for each recruitment cohort summarized in Table 1. Nearly half of SRPs reported learning about the study from a brochure or advertisement, whereas the majority of ARPs reported referral from another research study at the same institution. Twenty-two participants were missing information about how they learned about the study and excluded from cohort comparisons. Compared with participants who were part of the ARP and SRP cohorts, the group of participants missing recruitment information was more likely to self-identify as African American (48% versus 21%, *P* < 0.001) and to have enrolled at the Howard University site (45% versus 22%, *P* = 0.011). They were less likely to have a college degree (45% versus 69%, *P* = 0.023), to have household incomes of over $70,000 (29% versus 54%, *P* = 0.047) or to have enrolled at the Case Western Reserve site (5% versus 24%, *P* = 0.034).

Table 2 summarizes the demographic characteristics of the SRP and ARP cohorts. SRPs tended to be younger, less likely to self-identify as African American, and had higher household incomes than ARPs. They were also more likely to have enrolled at the Case Western Reserve, Weill School of Medicine, and University of Michigan sites and less likely to have enrolled at the Boston University site. SRPs comprised a greater percentage of the third study trial and a lower percentage of the second study trial. Finally, SRPs were more likely to be employed than ARPs, although retirees largely drove this difference: 39% of ARPs reported being retired compared with 22% of SRPs (*P* < 0.001). Additional within-trial analyses showed no differences between cohorts in randomization statuses, nor in the likelihood of having an AD-affected FDR (ARP, 63%; SRP, 69%; *P* = 0.293). Cohorts also did not differ by gender-age or gender-race combinations (*P* = 0.566 and 0.692, respectively).

**Table 2:** **Descriptive statistics (number and percentage) of REVEAL study participants by recruitment cohort**

**Characteristic**	**Actively recruited (n = 249)**	**Self-referred (n = 546)**	***P***
Age ≥60 years	147 (59.0%)	223 (40.8%)	**<0.001**
Male	87 (34.9%)	182 (33.3%)	0.666
Black/African American	86 (34.5%)	81 (14.8%)	**<0.001**
College degree or higher*	130 (66.3%)	339 (70.0%)	0.343
Household income ≥ $70 K*	83 (45.1%)	264 (57.1%)	**0.006**
Has an AD-affected first-degree relative (parent or sibling)	216 (86.7%)	458 (83.9%)	0.297
Has an AD-affected family member beyond first-degree relatives	106 (44.2%)	281 (51.6%)	0.056
Employed part/full time*	99 (50.5%)	323 (66.9%)	**<0.001**
ε4 carrier^†^	46 (33.6%)	144 (37.1%)	0.459
Site by referral cohort			**<0.001**
Boston University	122 (49.0%)	126 (23.1%)	
Case Western Reserve	46 (18.5%)	145 (26.6%)	
Howard University	63 (25.3%)	115 (21.1%)	
Weill School of Medicine	11 (4.4%)	84 (15.4%)	
University of Michigan	7 (2.8%)	76 (13.9%)	
Trial by referral cohort			**<0.001**
Second trial	160 (64.3%)	263 (48.2%)	
Third trial	89 (35.7%)	283 (51.8%)	

*Assessed during the telephone interview (196 actively recruited participants, 484 self-referred participants, 680 total).

^†^Determined through genotyping among participants who provided blood samples (137 actively recruited participants, 388 self-referred participants, 525 total). *P*-values are bolded where differences between cohorts were statistically significant.

SRPs scored higher than ARPs on scales of AD concern (3.5 versus 3.3, *P* = 0.039) and were more likely to declare interest in having a genetic test to assess AD risk (94% versus 89%) in bivariate analyses of data collected at enrollment, and scored higher on AD attentiveness (2.1 versus 1.9, *P* < 0.001) in bivariate analyses of data collected during the phone interview (Additional file 2). In addition, SRPs from the second trial scored higher than ARPs on perceived susceptibility, although differences were not noted in the third trial. Only differences in AD attentiveness (*P* < 0.001) and perceived AD susceptibility among participants from the second trial (*P* = 0.011) remained significant in analyses of participants who completed the pre-education questionnaire, however (Table 3).

**Table 3:** **Beliefs about Alzheimer’s disease and genetic testing within each recruitment cohort among participants who completed the pre-education questionnaire**

	**Actively recruited (n = 163)**	**Self-referred (n = 444)**		
**Continuous/ordinal measures (range)**	**Mean ± SD**	**Mean ± SD**	**∆ (95% CI)**	***P***
Perceived susceptibility, second trial (0–100)	51.5 ± 22.1	58.6 ± 20.8	7.1 (1.7 to 12.6)	0.011
Perceived susceptibility, third trial (0–100)	35.5 ± 25.3	34.0 ± 21.3	−1.5 (1.7 to 12.6)	0.664
Perceived seriousness (1–5)	3.1 ± 1.5	3.2 ± 1.5	0.1 (−0.2 to 0.4)	0.389
AD concern (1–5)	3.4 ± 0.8	3.5 ± 0.7	0.1 (0.0 to 0.2)	0.122
AD attentiveness (1–4)	1.9 ± 0.8	2.1 ± 0.8	0.2 (0.1 to 0.4)	**0.006**
Coping self-efficacy (0–100)	86.3 ± 19.4	86.0 ± 18.7	−0.3 (−3.9 to 3.2)	0.861
Perceived pros (1–5)	3.5 ± 0.8	3.5 ± 0.7	0.0 (−0.1 to 0.1)	0.949
Perceived cons (1–5)	1.9 ± 0.7	1.9 ± 0.7	−0.1 (−0.2 to 0.0)	0.228
Causal belief: genetics/heredity (1–5)	4.1 ± 0.9	4.1 ± 0.9	0.0 (−0.2 to 0.1)	0.785
Causal belief: lifestyle (1–5)	3.5 ± 1.2	3.4 ± 1.1	−0.1 (−0.3 to 0.1)	0.525
**Binary measures**	**n (%)**	**n (%)**	**OR (95% CI)**	***P***
Interest in genetic risk assessment	159 (97%)	434 (98%)	1.2 (0.4 to 3.4)	0.779
Expectation of reassurance	19 (12%)	65 (15%)	1.3 (0.7 to 2.3)	0.412
Expectation of aided decision making	21 (13%)	58 (13%)	1.0 (0.6 to 1.8)	0.940

Results are adjusted for demographic factors that varied by cohort (age, race, income, employment status, study site, and study trial). CI, confidence interval; OR, odds ratio.

### Study dropout

ARPs were more likely to drop out of the REVEAL study prior to genotype and AD risk disclosure than SRPs (41% versus 26%, *P* < 0.001). This association was still significant (odds ratio = 0.62, *P* = 0.011) in logistic regression analyses that controlled for all data collected at intake (that is, race, age, gender, interest, AD concern, perceived seriousness, perceived susceptibility) and study-specific factors (trial number, site). However, ARPs were no more likely than SRPs to drop out once they completed the pre-education questionnaire in unadjusted analyses (16% versus 13%, *P* = 0.286) or adjusted analyses (*P* = 0.423).

Across cohorts, those who received genetic risk information were more educated (*P* < 0.001), had higher incomes (*P* < 0.001), and were less likely to identify as female (*P* = 0.015) or African American (*P* < 0.001) than participants who did not. Those who received test results were also more likely to have been enrolled at the Weill School of Medicine and University of Michigan sites (*P* < 0.001; Additional file 3). Stratified analyses suggested that younger participants, lower income, African American race and site may have predicted dropout only among the SRP cohort, although no interaction effects between those factors and recruitment cohort were observed (all *P* > 0.110). The association between education and test uptake was stronger among SRPs than ARPs (*P* = 0.031), but no other interactions between recruitment cohort and demographic factors were observed with respect to test follow-through. Bivariate analyses also showed that individuals receiving genetic risk disclosure had greater interest in having a genetic risk assessment (*P* < 0.001), perceived fewer cons of testing (*P* < 0.001), and had greater coping self-efficacy than participants who dropped out (*P* < 0.001). Stratified analyses showed that the association between perceived cons and study dropout was borderline non-significant among ARPs (*P* = 0.058), but were otherwise consistent with aggregated data (Additional file 4).

### Behavioral responses

Overall, 33% of participants reported making a change to at least one of the queried health behaviors at the 6 week follow-up, and 55% reported making a change at the 12 month follow-up. Forty-seven percent reported intention to change health behaviors in the future. Across behaviors, recruitment mechanism was not associated with likelihood of reporting a behavior change (*P* = 0.319 at 6 weeks, *P* = 0.210 at 12 months) or intentions to change behavior (*P* = 0.719). Analyses of specific behaviors found that self-referred participants were more likely than ARPs to report changes to exercise at 12 months (35% versus 25%, *P* = 0.032). No other differences between recruitment cohorts were noted on changes or plans to change health behaviors.

**Figure 1:**
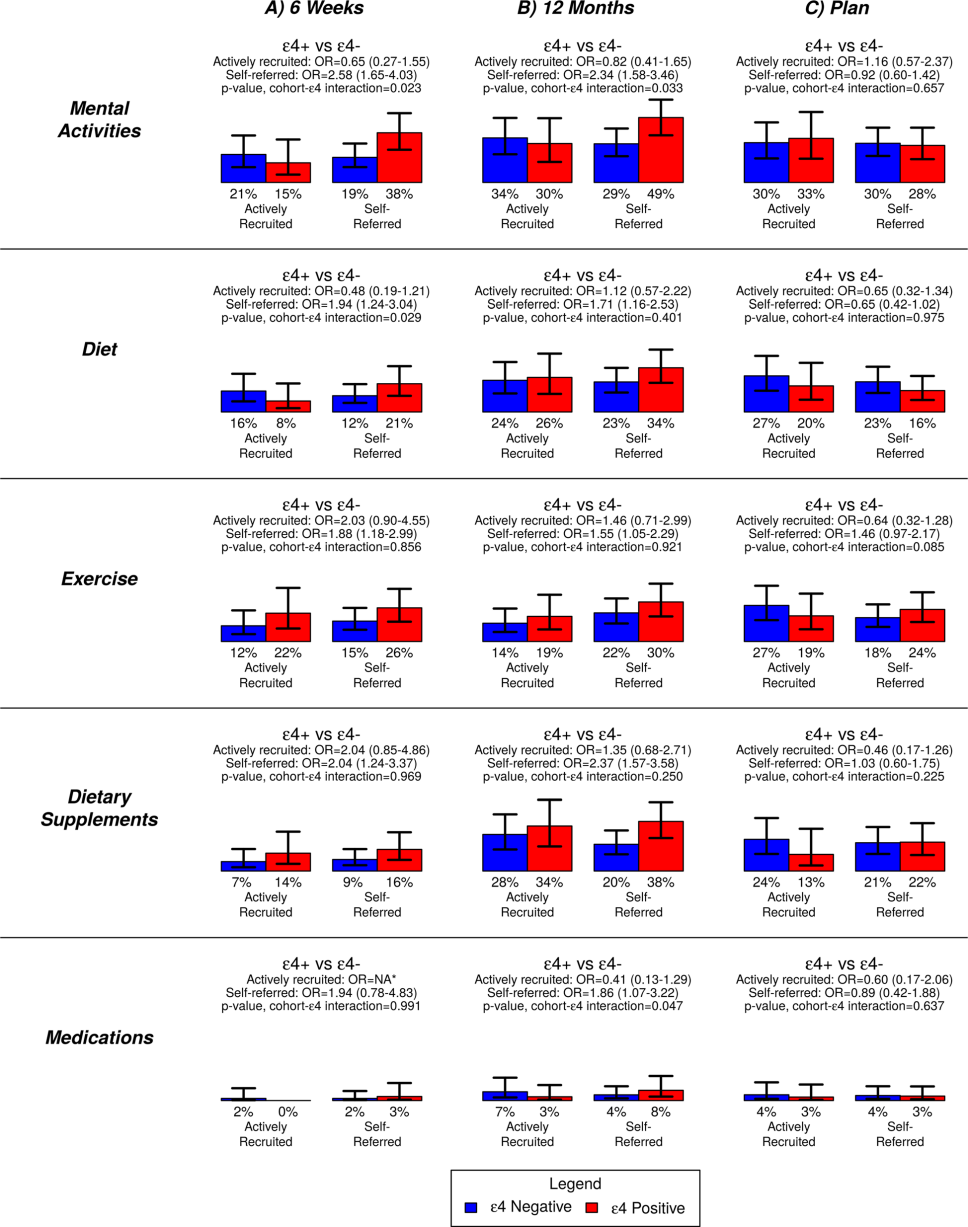
**Percentages of each recruitment cohort reporting changes to health behaviors, stratified by** ***APOE*** **status.** Analyses examine changes reported **(A)** 6 weeks and **(B)** 12 months after genetic risk disclosure, as well as **(C)** plans to change in the future. Analyses control for demographic and psychological characteristics that varied by cohort (age, race, income, employment status, study round, randomization status, site, perceived susceptibility and AD attentiveness). Error bars show 95% confidence intervals. Asterisks indicate an odds ratio (OR) cannot be calculated because no ARPs who were ε4-negative reported changes to medications.

Secondary analyses showed that the impact of genetic risk status on certain behavior changes differed by recruitment cohort, however. As Figure 1 shows, ε4-positive participants were more likely than ε4-negative participants to report changes at 6 weeks to mental activities and diet, but only if they had self-referred to the study (*APOE*-cohort interaction: *P* = 0.023 and 0.029, respectively), although only differences in changes to mental activities persisted through the 12 month follow-up. An interaction effect between ε4 status and recruitment cohort was also observed on changes to medications at 12 months (*P* = 0.047). Ironically, while nearly identical percentages of SRPs and ARPs who were ε4-negative discussed prevention strategies with the study clinician during the disclosure session (25% and 23%, respectively; *P* = 0.774), trends among ε4 carriers were opposite than anticipated: 38% of the self-referred cohort discussed prevention options compared with 54% of the actively recruited cohort (*P* = 0.053). Of additional note, the SRP cohort was no more likely to continue behavior changes reported at 6 weeks than the ARP cohort, even in analyses that examined interactions between cohort and *APOE* status (all *P* > 0.05).

**Figure 2:**
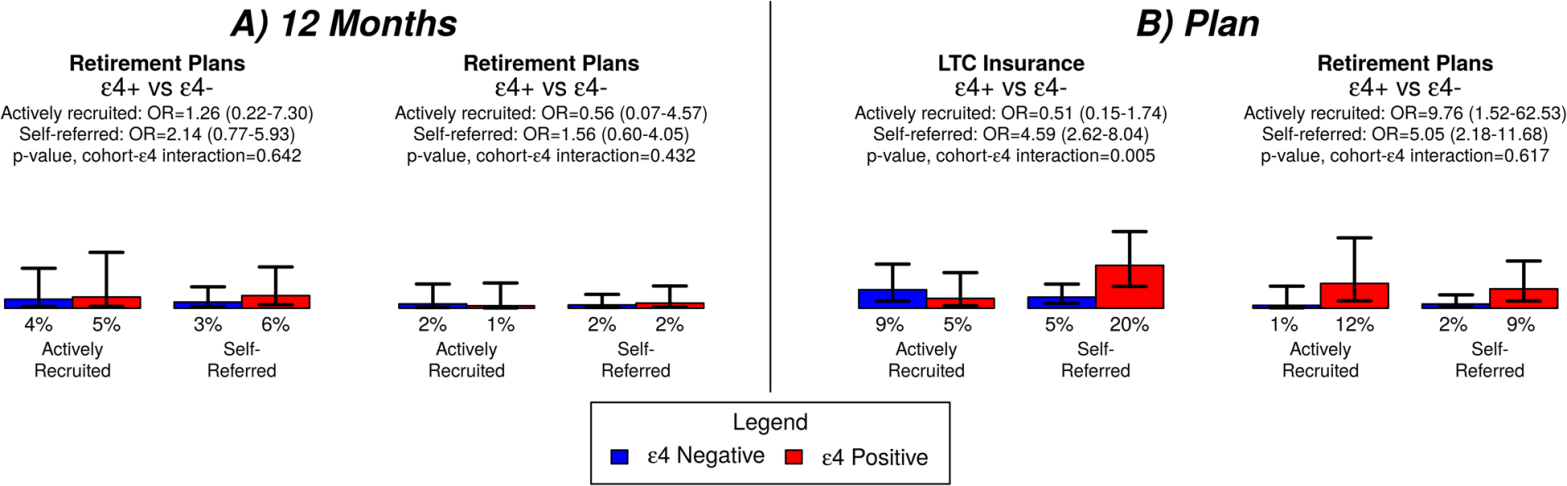
**Percentages of each recruitment cohort reporting changes to advance planning outcomes, stratified by** ***APOE*** **status.** Analyses examine changes reported **(A)** 12 months after genetic risk disclosure, as well as **(B)** plans to change in the future. Analyses control for demographic and psychological characteristics that varied by cohort (age, race, income, employment status, study round, randomization status, site, perceived susceptibility and AD attentiveness). Error bars show 95% confidence intervals.

Similarly, 6% of participants reported a change to either long-term care (LTC) insurance coverage or retirement plans at the 12 month follow-up, while 16% reported intentions to change advance planning. No direct associations with self-referral were observed on either LTC insurance coverage or retirement plans. An interaction effect was observed (*P* = 0.005): self-referred ε4-positive participants were more likely than ε4-negative participants to report intentions to change LTC coverage, but no differences were noted among ARPs (Figure 2). No associations were noted on retirement plans, except greater intentions to change among ε4-positive participants compared with ε4-negative participants, regardless of recruitment cohort (*P* < 0.001).

## Discussion and conclusions

This paper addresses the implications of different study recruitment strategies on responses to genetic risk information, using the model of *APOE* genotype disclosure for risk of AD. While prior REVEAL study analyses showed that ε4-positive participants were more likely than ε4-negative participants to report changes to health behaviors and LTC insurance coverage [3-6], these follow-up analyses show that such responses often depended upon whether or not an individual had proactively sought testing. ε4-positive participants were more likely to report changes to mental activities, short-term changes to diet, and plans to change LTC insurance coverage than ε4-negative participants, but only if individuals had self-referred into the study.

Our data do not fully explain why this difference is present. Self-referred participants in the REVEAL study may be more prepared to act on indications of increased risk for AD. While few differences were observed at baseline on beliefs about AD and attitudes about testing, such factors represent only a fraction of the psychosocial determinants that facilitate or inhibit behavior change. Many have argued that risk assessment interventions fall short by ignoring more critical determinants of behavior change, especially motivation, behavioral capacity and self-efficacy [32]. Supporting this notion was the finding that self-referred individuals were less likely to discuss prevention strategies during genetic risk disclosure, yet more likely to respond to indications of increased genetic risk. Interventions that hope to use genetic risk information to motivate changes to lifestyles and other behaviors may benefit from more careful consideration of the readiness of individuals to enact such changes [33]. Practitioners and researchers must also be sensitive to the potential for studies of genetic risk information to inflate its ability to motivate health behavior changes by enrolling self-referred populations. Conclusions from such studies may not apply to populations that are less prone to proactively seek out genetic susceptibility testing.

The implications of these findings are timely, given advancements in technology to analyze genomes and provide genetic susceptibility information. In particular, great attention has been given to whether and how findings from genomic sequencing and genetic research should be disclosed, including calls to improve understanding of the impact of disclosing unsolicited genomic risk information derived as incidental or secondary findings [34-37]. In addition, questions persist about the utility of direct-to-consumer genetic testing [38-40], which has overwhelmingly relied on passive marketing strategies that favor self-referral [41] (and companies have increasingly incorporated *APOE* into their testing panels) [42]. The results from our analyses underscore how the impact of genomic information disclosure on lifestyles and advance planning decisions may be very different under one model of service provision than another.

Although the focus of these analyses has been on changes to health behaviors and advance planning, a number of additional findings are of interest. As seen in the first REVEAL study trial, SRPs tended to be younger and had higher household incomes than ARPs [16]. They were also more likely to be employed, more interested in testing, and had greater attentiveness about AD than ARPs. Of particular note - and contrary to findings from the first REVEAL study trial, which enrolled ARPs exclusively through research registries - was how SRPs had less than half the percentage of African Americans at intake. Prior research has found African Americans to have less interest in hypothetical genetic testing for AD [43] and actual genetic testing for susceptibility to other diseases [44]. This finding underscores the need for aggressive outreach if researchers hope to diversify the samples enrolling in genetic susceptibility testing research, while also suggesting caution when interpreting racial differences in behavioral outcomes when traditionally underrepresented groups are actively recruited. SRPs were also more likely to progress from enrollment to testing without dropping out, as found in analyses from the first REVEAL study trial [16]. Studies favoring active recruitment strategies should plan accordingly for greater participant attrition.

A number of limitations should be mentioned. Differences in recruitment strategies also introduced differences in sampling frames, complicating cohort comparisons. Study measures had several limitations, including that behavioral outcomes were single-item self-reports of behavior change and participant to social desirability biases [45]. Study participants, whether self-referred or actively recruited, were highly educated volunteers with high socioeconomic status, and were positively inclined toward genetic testing by virtue of their participation. As research subjects, test results were omitted from medical records, providing protections from discrimination that clinical testing may not offer [24]. Statistical significance was set at *P* = 0.05 despite multiple comparisons, and findings about changes to health behaviors would not have been statistically significant at a more conservative value of *P* = 0.01. Analyzed data were collected over a 6-year period that overlapped with advances in AD prevention research [46], the emergence of direct-to-consumer genetic testing [40], and the enactment of legislation prohibiting genetic discrimination [24] which could have changed attitudes towards genetic susceptibility testing for AD. Finally, the REVEAL study focused on single gene testing for AD risk, and findings may not generalize well to contexts where multiple genes and multiple diseases are addressed.

Nevertheless, these analyses demonstrate how different models of recruitment for providing genetic risk information affect who pursues testing and how they respond to the information they receive. Policymakers and clinicians will need to be sensitive to the ways genetic risk information can be obtained as they weigh its ability to improve preventive behaviors and public health.

## Additional files

Additional file 1:
**The education brochures used in the second and third REVEAL study trials.**
Additional file 2:
**Bivariate comparisons of recruitment cohorts.**
Additional file 3:
**Study dropout by demographic factors.**
Additional file 4:
**Study dropout by AD and genetic testing beliefs.**
Additional file 5:
**Additional REVEAL Study Group members.**

## Abbreviations

AD: Alzheimer’s disease
ARP: actively recruited participant
FDR: first degree relative
LTC: long-term care
REVEAL: Risk Evaluation and Education for Alzheimer’s Disease
SRP: self-referred participant
