# Supplementary material for: Associations between self-referral and health behavior responses to genetic risk information
Source: Genome Med. 2015 Jan 31;7(1):10. doi: 10.1186/s13073-014-0124-0 (PMC4311425; doi:10.1186/s13073-014-0124-0)

Supplemental Figure 1. Educational brochures used in the 2nd and 3rd REVEAL Study trials

Understanding Your Risk of Alzheimer's Disease

Alzheimer's disease is a progressive brain disorder that ultimately impairs a person's ability to carry out daily activities; it is the most common cause of memory and language problems (sometimes referred to as "senility" or "dementia") among older people. Although much is known about the disease's biology, and treatments are available for some of its symptoms, there is currently no cure or prevention for Alzheimer's disease. An estimated four million Americans have Alzheimer's disease—a number that is projected to grow to over 13 million by 2050.

Alzheimer's Risk Factors

The general population's risk of developing Alzheimer's disease is about 10–15%. This means that for every group of 100 people, on average, 10–15 of them will develop Alzheimer's disease at some point in their life. Some people have a higher or lower risk than the general population. One or more of the following factors can elevate a person's Alzheimer's disease risk above that of the general population:

- ↗ Getting older
- ↗ Being a woman
- ↗ Being African American
- ↗ Having a parent, brother, or sister with Alzheimer's disease
- ↗ Inheriting a specific form of the APOE gene

However, it is important to remember that if you have one—or even all—of these characteristics, you still may never get Alzheimer's disease.

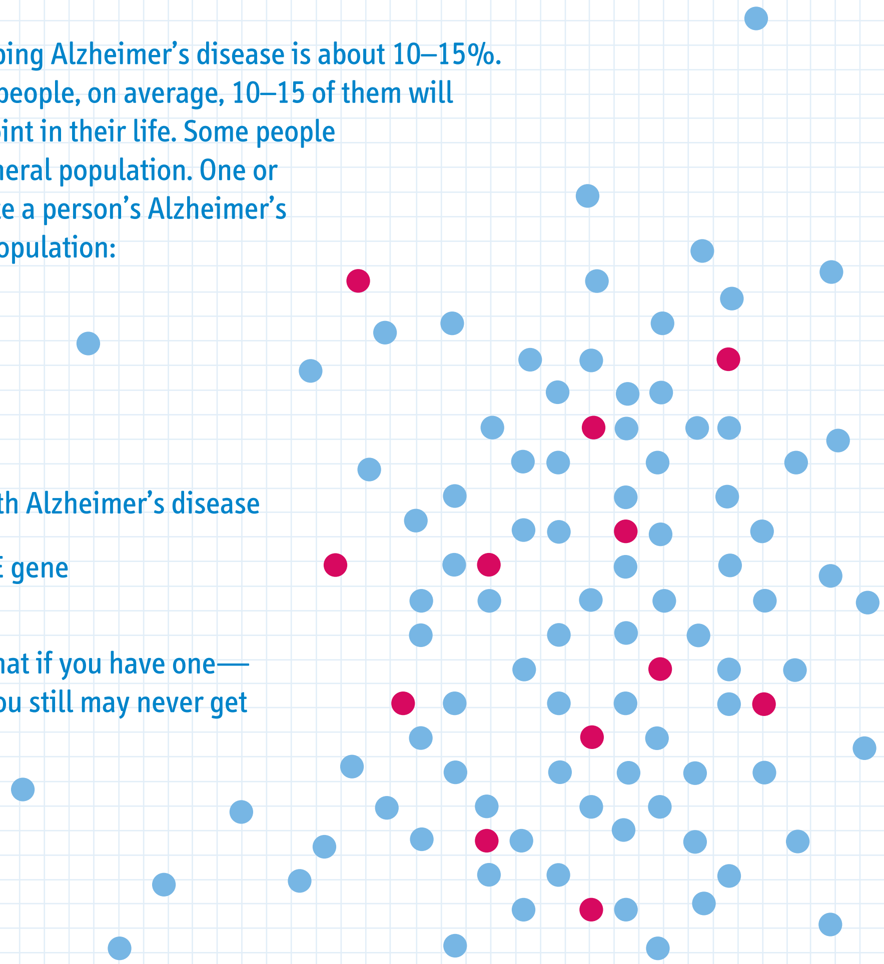

Alzheimer's Disease and the APOE Gene

Inheriting a specfic form of the APOE gene can increase the risk of getting Alzheimer's disease. The role of the APOE gene in Alzheimer's disease is still being studied. Some studies have shown that it may be related to other conditions in addition to Alzheimer's disease.

We do know that the APOE gene comes in three different forms: E2, E3, and E4. Every person has two copies of the APOE gene—one inherited from each parent. Because there are three different forms of the APOE gene and there are two APOE genes in every person, an individual posesses one of six unique APOE combinations (pictured below).

If an individual has one or two copies of the E4 form of the APOE gene, it increases his or her risk of developing Alzheimer's disease. However, this does not mean that he or she will definitely get Alzheimer's disease.

APOE Genetic Testing

As part of your risk assessment, we provide APOE testing. There are three basic steps to APOE testing. First, you will meet with a genetic counselor to review any questions or concerns about having an Alzheimer's disease risk assessment, including APOE testing. Next, you will provide a small blood sample for APOE testing. Finally, you will meet with a clinician to learn and discuss your test result and risk assessment. Test results are typically available within a few weeks of the blood draw.

E2

E3

E4

There are three different forms of the APOE gene—E2, E3, and E4.

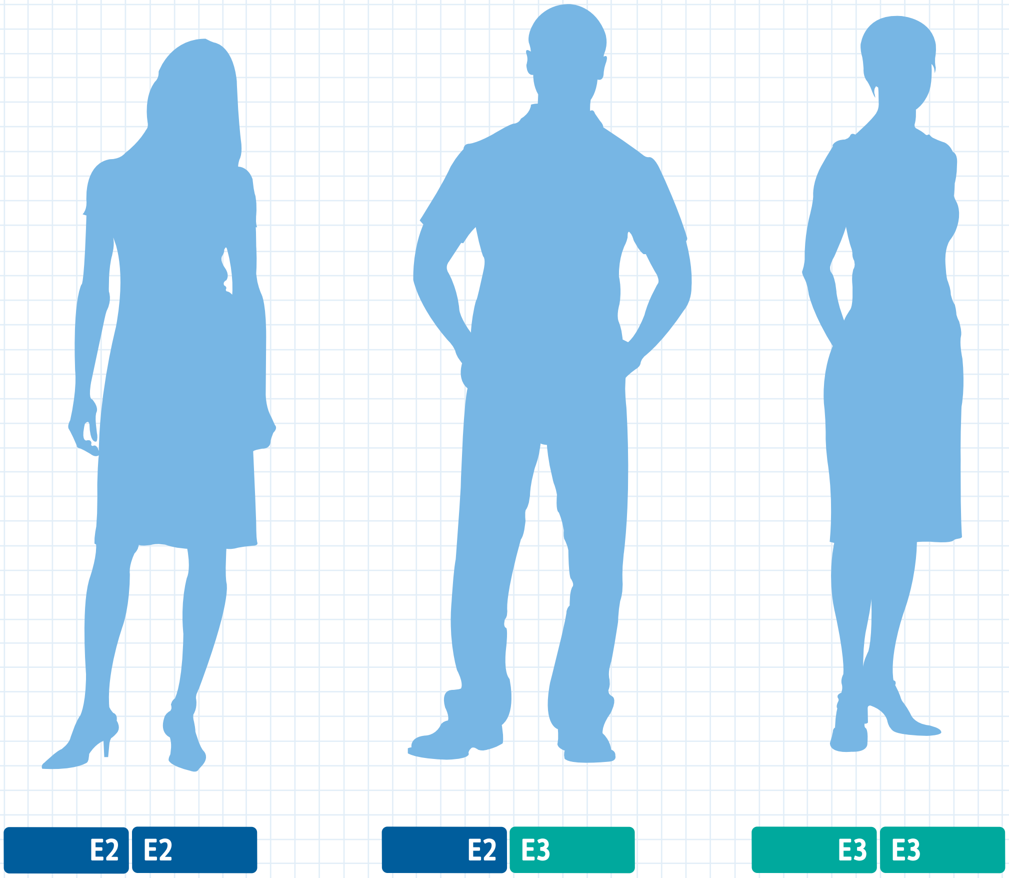

Understanding Your Risk Assessment

You will be given an estimate of your risk of developing Alzheimer's disease by the time you are 85 years old. Depending on your risk factors, you will be given a risk number between approximately 15% to 75%. Your risk estimate will also be shown on a graph, similar to that pictured below.

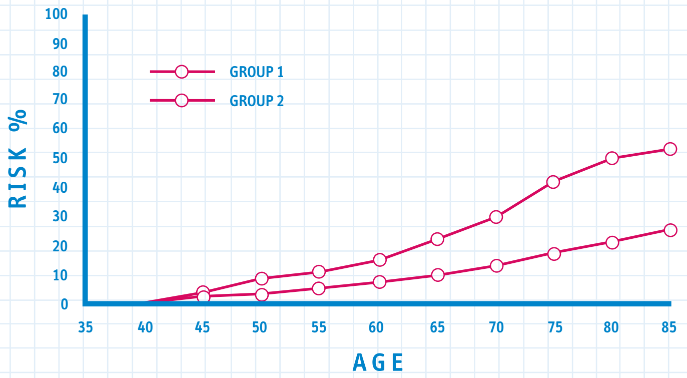

The characteristics taken into account in the risk assessment include your age, gender, race, APOE test result, and whether or not you have a parent, brother, or sister with Alzheimer's disease.

We are still learning about many other genetic and non-genetic factors that are involved in the development of Alzheimer's disease. As scientists learn more about what causes Alzheimer's disease, this new information may alter your risk assessment.

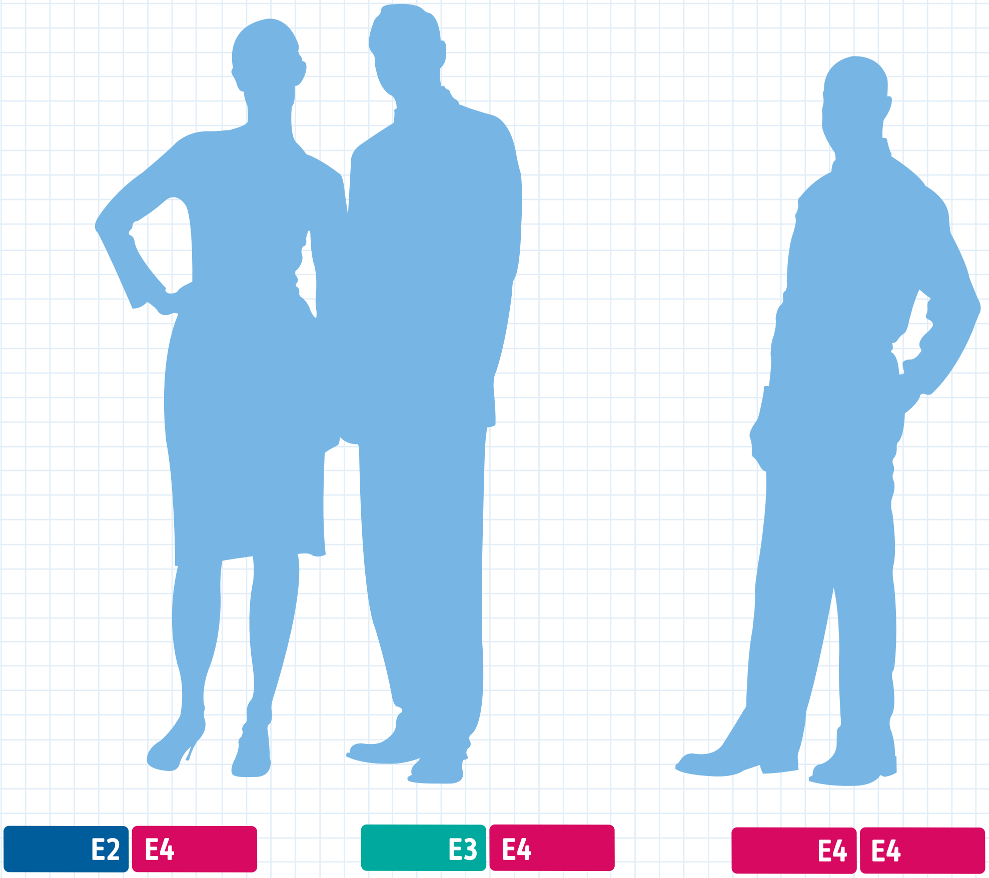

There are six possible combinations of the three APOE forms. These combinations are called **genotypes.**

Issues to Consider

You may want to consider several issues before having a risk assessment for Alzheimer's disease. Your risk profile is only an interpretation based on our current knowledge and will not give you a simple "yes" or "no" answer, nor will it indicate at what age Alzheimer's disease may develop. There are no proven ways to prevent Alzheimer's disease from developing.

Risk assessment with APOE testing has some limitations:

↗ You may find it harder to cope with your concerns about developing Alzheimer's disease after having your risk assessment

↗ Because the risk assessment is based on complex information, it is possible that you or your loved one may misinterpret the results, causing undue stress or false reassurance about your chances of developing Alzheimer's disease

↗ Confidentiality laws protect APOE test results generated for research purposes. However, if you tell others about your results, there is no guarantee that your results will remain confidential

↗ Employers or insurance companies could ask you about your risk information and use it to deny insurance coverage or change your policy rates, although it is not a common practice.

There may also be some benefits to receiving your risk estimate:

↗ It may encourage you to stay abreast of new developments in Alzheimer's disease treatment and prevention, and may motivate you to engage in activities that might help prevent or delay the onset of Alzheimer's disease

↗ It may satisfy your curiosity about your chances of developing the disease

↗ You may use your risk assessment to help make long term decisions

↗ Receiving a lower risk estimate may reduce your anxiety about developing the disease.

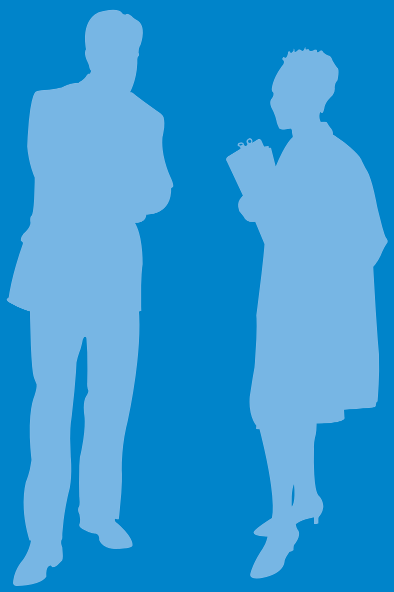

Resources

Contact the following organizations to learn more about Alzheimer's disease.

Alzheimer's Association
800-272-3900
alz.org

Alzheimer's Disease Education & Referral Center (ADEAR), a service of the National Institute on Aging (NIA).
800-438-4380
alzheimers.org

DESIGN © 2004 SCHWADESIGN, INC.

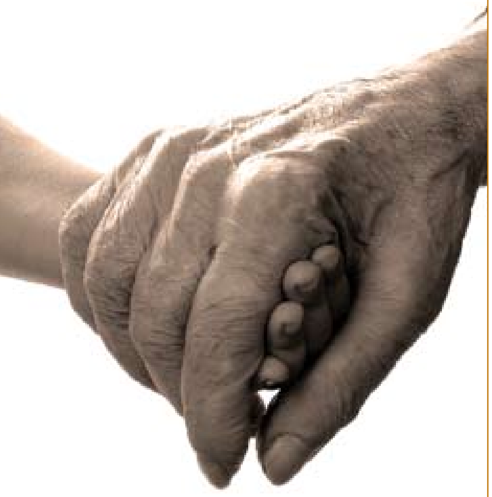

Understanding Your Risk of Alzheimer's Disease

Alzheimer's disease is a brain disease that is the most common cause of memory and language problems in older people. It can impair a person's ability to carry out daily activities. Although we are learning more about the cause of the disease, and treatments are available for some of its symptoms, there is currently no cure or prevention for Alzheimer's disease. An estimated 5 million Americans have Alzheimer's disease - a number that is projected to grow to over 13 million by 2050.

The general population's lifetime risk of developing Alzheimer's disease is about 12%.

Who is at Risk for Alzheimer's Disease?

The risk of developing Alzheimer's disease in the general population is about 12%. This means that for every group of 100 people, 12 of them will develop Alzheimer's disease at some point in their life.

Studies have shown that certain groups of people have higher than average rates of Alzheimer's disease, although the reasons for this are still to be determined. Research suggests that the following groups are at elevated risk of Alzheimer's disease:

- Older adults
- Women
- African Americans
- People with a parent or sibling affected by Alzheimer's disease
- People with a specific form of the APOE gene
- People with existing memory problems

However, it is important to remember that even if you are a member of one or more of these groups, you may still never develop Alzheimer's disease.

Alzheimer's Disease and the APOE Gene

Inheriting a specific form of the APOE gene can increase the risk of getting Alzheimer's disease. The APOE gene's specific role in Alzheimer's disease is still being studied. Some studies have shown that it may be related to other conditions in addition to Alzheimer's disease.

We do know that the APOE gene comes in three different forms: e2, e3, and e4. Every person has two copies of the APOE gene—one inherited from each parent. Thus, an individual can have one of six possible combinations of the APOE gene (pictured below).

An individual who carries one or two copies of the e4 form of the APOE gene is at increased risk of developing Alzheimer's disease.

APOE Genetic Testing

As part of your risk assessment, we provide APOE testing. There are three basic steps to APOE testing. First, you meet with a genetic counselor to review your family history and any questions or concerns that you have. Next, you provide a small blood sample for APOE testing. Test results are typically available within a few weeks of the blood draw. Finally, you meet with a clinician to learn and discuss your test result and risk assessment.

There are three different forms of the APOE gene—e2, e3, and e4.

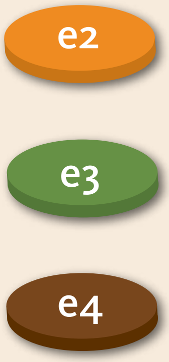

There are six possible combinations of the three APOE forms. These combinations are called genotypes.

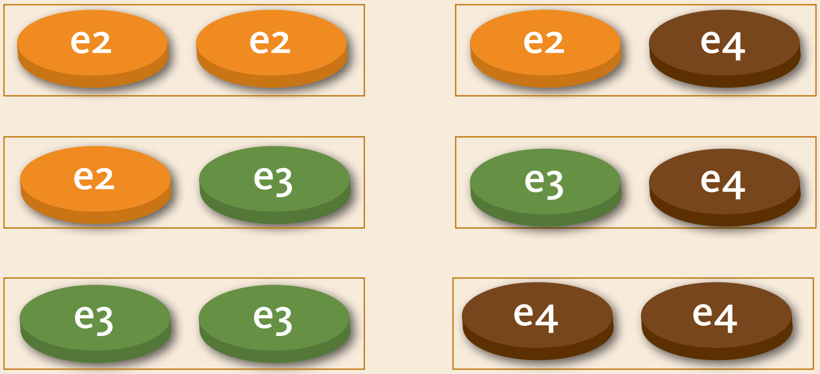

Understanding Your Risk Assessment

You will be given an estimate of your risk of developing Alzheimer's disease by the time you are 85 years old. Depending on your risk factors, you will be given a risk number ranging from 10% to 77%. Your risk will also be shown on a graph, similar to the one pictured below, to help illustrate your risk according to your age.

The characteristics taken into account in the risk assessment include your age, gender, ethnicity, APOE test result, whether or not you have a parent, brother, or sister with Alzheimer's disease, and whether or not you have existing memory problems. We are still learning about many other genetic and non-genetic factors that are involved in the development of Alzheimer's disease. As scientists learn more about what causes Alzheimer's disease, this new information may alter your risk assessment.

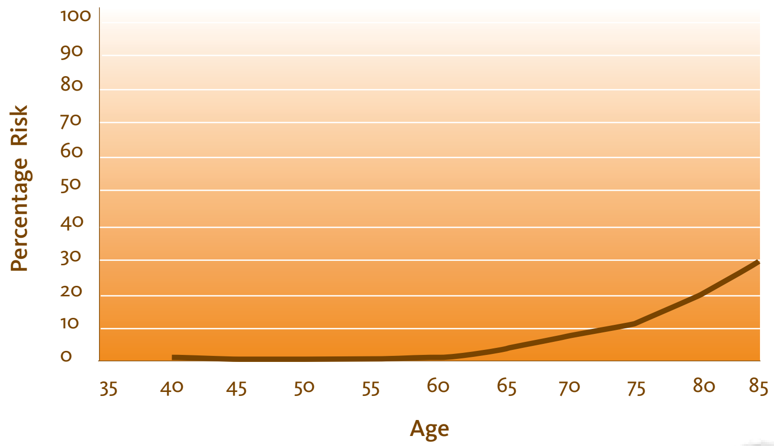

Inheriting the e4 form of the APOE gene can increase the risk of getting Alzheimer's disease.

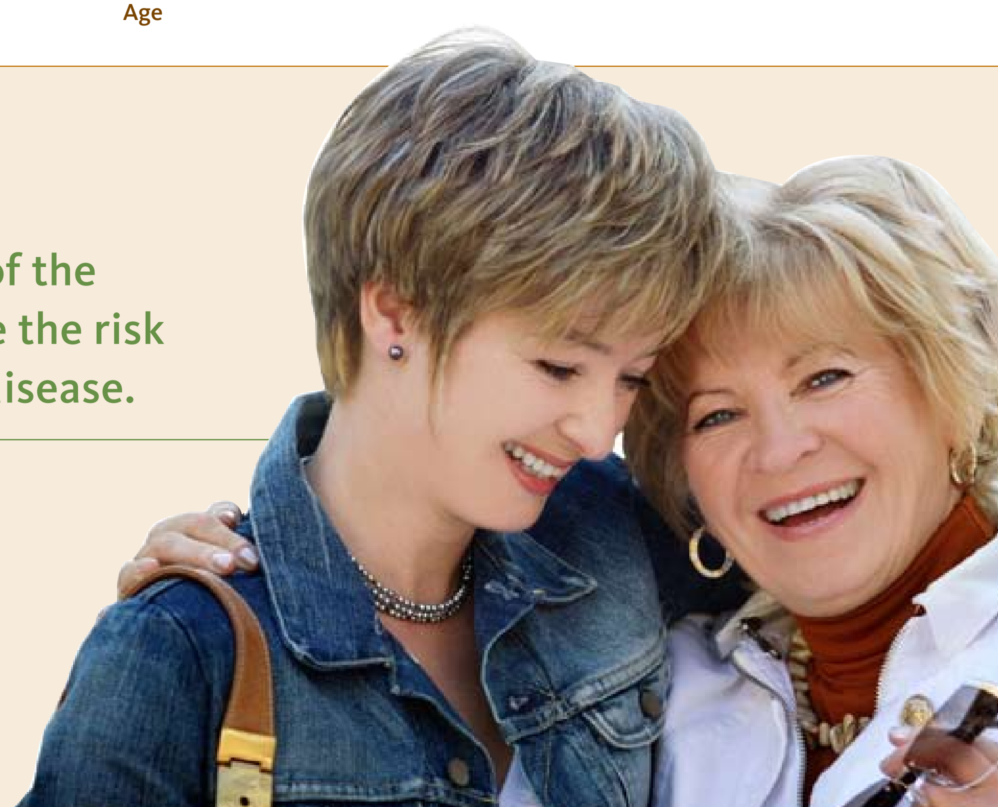

Factors to Consider

You may want to consider several factors before having a risk assessment for Alzheimer's disease. Certain limitations are inherent in the process. Your risk profile is an interpretation based on our current knowledge and will not give you a simple "yes" or "no" answer, nor will it indicate at what age Alzheimer's disease may develop. There are no proven ways to prevent Alzheimer's disease from developing. Other factors to take into account include:

- You may find it harder to cope with your concerns about developing Alzheimer's disease after having your risk assessment
- Because the risk assessment is based on complex information, you or your loved one may misinterpret the results, causing undue stress or false reassurance about your chances of developing Alzheimer's disease
- Generally, confidentiality laws protect APOE test results generated for research purposes. However, if you tell others about your results, there is no guarantee that your results will remain confidential
- Although it is not a common practice, employers or insurance companies could ask you about your risk information and use it to deny insurance coverage or change your policy rates.

There are also some advantages to receiving your risk estimate. Consider that:

- It may encourage you to stay abreast of new developments in Alzheimer's disease treatment and prevention and make long term planning decisions,
- It may satisfy your curiosity about your chances of developing the disease,
- You may be motivated by your risk estimate to engage in activities that might help prevent or delay the onset of Alzheimer's disease, and
- Receiving a lower risk estimate may reduce your anxiety about developing the disease.

Resources

Contact the following organizations to learn more about Alzheimer's disease.

Alzheimer's Association
800-272-3900
http://www.alz.org

Alzheimer's Disease Education & Referral Center (ADEAR),
a service of the National Institute on Aging (NIA).
800-438-4380
http://www.alzheimers.org

This brochure was produced as part of the Risk Evaluation and Education for Alzheimer's Disease (REVEAL) Study based at Boston University Medical Center
http://www.bu.edu/alzresearch

This research was funded by the Ethical, Legal and Social Implications branch of the National Human Genome Research Institute (NHGRI)
http://www.genome.gov

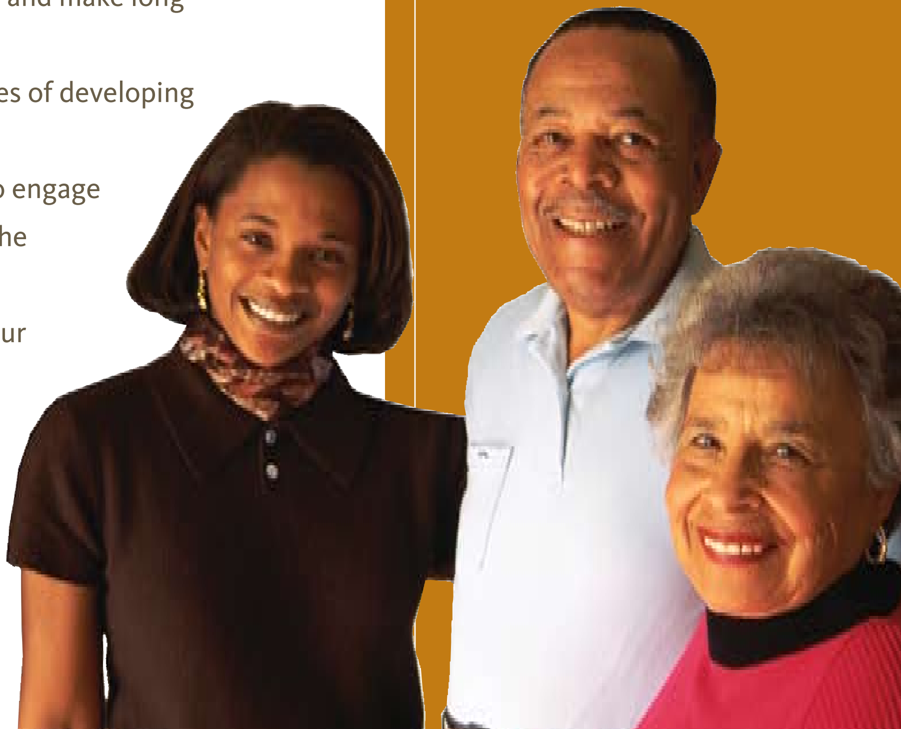

Supplement: Additional file 1: — The education brochures used in the second and third REVEAL study trials. [file 13073_2014_124_MOESM1_ESM.pdf]
