# Supplementary material for: Associations between self-referral and health behavior responses to genetic risk information
Source: Genome Med. 2015 Jan 31;7(1):10. doi: 10.1186/s13073-014-0124-0 (PMC4311425; doi:10.1186/s13073-014-0124-0)
Supplement: Additional file 2: — Bivariate comparisons of recruitment cohorts. [file 13073_2014_124_MOESM2_ESM.docx]

Supplemental Table 1. Beliefs about AD and genetic testing within each recruitment cohort among participants who completed the pre-education questionnaire. Results are unadjusted. Differences between cohorts may not equal differences between columns exactly due to rounding.

|  | **Actively Recruited**  **(n=163)** | **Self-Referred**  **(n=444)** |  |  |
| --- | --- | --- | --- | --- |
| *Continuous/Ordinal Measures (range)* | *Mean ± SD* | *Mean ± SD* | *∆ (95% CI)* | *p* |
| Perceived susceptibility, 2^nd^ trial (0-100) | 52.3 ± 22.1 | 58.7 ± 20.8 | 6.4 (1.4 to 11.5) | 0.013 |
| Perceived susceptibility, 3^rd^ trial (0-100) | 36.0 ± 25.3 | 33.5 ± 21.3 | -2.5 (-8.9 to 3.8) | 0.436 |
| Perceived seriousness (1-5) | 3.1 ± 1.4 | 3.2 ± 1.4 | 0.1 (-0.2 to 0.3) | 0.551 |
| AD concern (1-5) | 3.4 ± 0.8 | 3.5 ± 0.7 | 0.1 (0.0 to 0.2) | 0.123 |
| AD attentiveness (1-4) | 1.9 ± 0.8 | 2.1 ± 0.8 | 0.3 (0.1 to 0.4) | **<0.001** |
| Coping self-efficacy (0-100) | 86.1 ± 18.3 | 86.1 ± 18.3 | 0.0 (-3.3 to 3.3) | 0.982 |
| Perceived pros (1-5) | 3.6 ± 0.7 | 3.5 ± 0.7 | 0.0 (-0.2 to 0.1) | 0.708 |
| Perceived cons (1-5) | 1.9 ± 0.7 | 1.9 ± 0.7 | -0.1 (-0.2 to 0.1) | 0.315 |
| Causal belief: hereditary/genetics (1-5) | 4.0 ± 0.8 | 4.1 ± 0.8 | 0.1 (-0.1 to 0.2) | 0.260 |
| Causal belief: lifestyle (1-5) | 3.5 ± 1.1 | 3.5 ± 1.1 | 0.0 (-0.2 to 0.2) | 0.979 |
|  |  |  |  |  |
| *Binary Measures* | *n (%)* | *n (%)* | *OR (95% CI)* | *p* |
| Interest in genetic risk assessment | 156 (96%) | 432 (97%) | 1.6 (0.6 to 4.2) | 0.323 |
| Expectation of reassurance | 26 (16%) | 86 (19%) | 1.3 (0.8 to 2.0) | 0.337 |
| Expectation of aided decision making | 28 (17%) | 76 (17%) | 1.0 (0.6 to 1.6) | 0.986 |
