# Supplementary material for: Associations between self-referral and health behavior responses to genetic risk information
Source: Genome Med. 2015 Jan 31;7(1):10. doi: 10.1186/s13073-014-0124-0 (PMC4311425; doi:10.1186/s13073-014-0124-0)
Supplement: Additional file 3: — Study dropout by demographic factors. [file 13073_2014_124_MOESM3_ESM.docx]

Supplemental Table 2. Bivariate associations between demographic characteristics and study dropout before results disclosure, stratified by recruitment cohort. Associations that are significant at p<0.05 are shown in bold font.

|  | **Actively Recruited Participants** | | |  | **Self-Referred Participants** | | |  | **Total** | | |
| --- | --- | --- | --- | --- | --- | --- | --- | --- | --- | --- | --- |
|  | **Dropped (n=112)** | **Disclosed (n=137)** | **p** |  | **Dropped (n=158)** | **Disclosed (n=388)** | **p** |  | **Dropped (n=270)** | **Disclosed (n=525)** | **p** |
| *Continuous/ordinal variables: mean (sd) unless noted* | | | | |  |  |  |  |  |  |  |
| Age | 61.7 (12.0) | 61.7 (11.8) | 0.982 |  | **54.3 (13.0)** | **57.0 (11.4)** | **0.015** |  | 57.4 (13.1) | 58.2 (11.7) | 0.338 |
| Years of education* | 15.7 (3.4) | 16.2 (2.8) | 0.276 |  | **15.3 (2.6)** | **16.5 (2.3)** | **<0.001** |  | **15.5 (3.0)** | **16.5 (2.5)** | **<0.001** |
| Median household income* | $50-69K | $50-69K | 0.097 |  | **$50-69K** | **$70-99K** | **<0.001** |  | **$50-69K** | **$70-99K** | **<0.001** |
| Number of AD-affected relatives | 1.6 (1.1) | 1.6 (1.2) | 0.853 |  | 1.7 (1.1) | 1.8 (1.3) | 0.625 |  | 1.7 (1.1) | 1.7 (1.2) | 0.608 |
|  |  |  |  |  |  |  |  |  |  |  |  |
| *Categorical variables: n (%)* | | |  |  |  |  |  |  |  |  |  |
| Male | 34 (30.4%) | 53.0 (38.7%) | 0.170 |  | **42 (26.6%)** | **140 (36.1%)** | **0.033** |  | **76 (28.1%)** | **193 (36.8%)** | **0.015** |
| Black/African American | **46 (41.1%)** | **40.0 (29.2%)** | **0.050** |  | **36 (22.8%)** | **45 (11.6%)** | **0.001** |  | **82 (30.4%)** | **85 (16.2%)** | **<0.001** |
| Employed part/full time* | 26 (44.1%) | 73.0 (53.3%) | 0.236 |  | 63 (65.6%) | 260 (67.2%) | 0.772 |  | 89 (57.4%) | 333 (63.5%) | 0.167 |
| Site |  |  | 0.051 |  |  |  | **<0.001** |  |  |  | **<0.001** |
| Boston University | 59 (52.7%) | 63.0 (46.0%) |  |  | **31 (19.6%)** | **95 (24.5%)** |  |  | **90 (33.3%)** | **158 (30.1%)** |  |
| Case Western Reserve | 15 (13.4%) | 31.0 (22.6%) |  |  | **51 (32.3%)** | **94 (24.2%)** |  |  | **66 (24.4%)** | **125 (23.8%)** |  |
| Howard University | 34 (30.4%) | 29.0 (21.2%) |  |  | **53 (33.5%)** | **62 (16.0%)** |  |  | **87 (32.2%)** | **91 (17.3%)** |  |
| Weill School of Medicine | 2 (1.8%) | 9.0 (6.6%) |  |  | **10 (6.3%)** | **74 (19.1%)** |  |  | **12 (4.4%)** | **83 (15.8%)** |  |
| University of Michigan | 2 (1.8%) | 5.0 (3.6%) |  |  | **13 (8.2%)** | **63 (16.2%)** |  |  | **15 (5.6%)** | **68 (13.0%)** |  |
| Trial |  |  | 0.420 |  |  |  | 0.984 |  |  |  | 0.271 |
| 2nd Round | 75 (67.0%) | 85.0 (62.0%) |  |  | 76 (48.1%) | 187 (48.2%) |  |  | 151 (55.9%) | 272 (51.8%) |  |
| 3rd Round | 37 (33.0%) | 52.0 (38.0%) |  |  | 82 (51.9%) | 201 (51.8%) |  |  | 119 (44.1%) | 253 (48.2%) |  |

* Assessed during the telephone interview (196 actively recruited participants, 484 self-referred participants, 680 total)
