# Supplementary material for: Associations between self-referral and health behavior responses to genetic risk information
Source: Genome Med. 2015 Jan 31;7(1):10. doi: 10.1186/s13073-014-0124-0 (PMC4311425; doi:10.1186/s13073-014-0124-0)
Supplement: Additional file 4: — Study dropout by AD and genetic testing beliefs. [file 13073_2014_124_MOESM4_ESM.docx]

Supplemental Table 3. Bivariate associations between psychosocial characteristics and study dropout before results disclosure, stratified by recruitment cohort. Associations that are significant at p<0.05 are shown in bold font.

|  | **Actively Recruited Participants** | | |  | **Self-Referred Participants** | | |  | **Total** | | |
| --- | --- | --- | --- | --- | --- | --- | --- | --- | --- | --- | --- |
|  | **Dropped (n=112)** | **Disclosed (n=137)** |  |  | **Dropped (n=158)** | **Disclosed (n=388)** |  |  | **Dropped (n=270)** | **Disclosed (n=525)** |  |
| *Continuous/Ordinal Measures (range)* | *Mean ± SD* | *Mean ± SD* | *p* |  | *Mean ± SD* | *Mean ± SD* | *p* |  | *Mean ± SD* | *Mean ± SD* | *p* |
| Perceived AD susceptibility, 2^nd^ trial (0-100) | 48.8 ± 27.0 | 51.1 ± 22.2 | 0.564 |  | 56.4 ± 22.9 | 58.4 ± 20.6 | 0.483 |  | 52.6 ± 25.2 | 56.2 ± 21.4 | 0.130 |
| Perceived AD susceptibility, 3^rd^ trial (0-100) | 37.8 ± 27.0 | 35.9 ± 27.2 | 0.716 |  | 35.1 ± 20.5 | 32.3 ± 21.3 | 0.320 |  | 35.9 ± 19.5 | 33.0 ± 22.6 | 0.241 |
| Perceived AD seriousness (1-5) | 3.0 ± 1.6 | 3.2 ± 1.5 | 0.470 |  | 3.1 ± 1.5 | 3.2 ± 1.4 | 0.766 |  | 3.1 ± 1.5 | 3.2 ± 1.4 | 0.446 |
| AD concern (1-5) | 3.4 ± 1.0 | 3.3 ± 0.8 | 0.926 |  | 3.4 ± 0.8 | 3.5 ± 0.6 | 0.102 |  | 3.4 ± 0.9 | 3.5 ± 0.7 | 0.156 |
| AD attentiveness* (1-4) | 1.9 ± 0.8 | 1.9 ± 0.7 | 0.710 |  | 2.0 ± 0.8 | 2.1 ± 0.8 | 0.314 |  | 2.0 ± 0.8 | 2.1 ± 0.8 | 0.305 |
| Coping self-efficacy* (0-100) | 83.2 ± 20.0 | 87.3 ± 16.4 | 0.140 |  | **79.4 ± 24.2** | **87.2 ± 17.1** | **<0.001** |  | **80.8 ± 22.7** | **87.2 ± 16.9** | **<0.001** |
| Perceived pros^†^ (1-5) | 3.5 ± 0.9 | 3.6 ± 0.8 | 0.720 |  | 3.6 ± 0.8 | 3.5 ± 0.7 | 0.489 |  | 3.6 ± 0.8 | 3.5 ± 0.7 | 0.724 |
| Perceived cons^†^ (1-5) | 2.2 ± 0.7 | 1.9 ± 0.7 | 0.058 |  | **2.1 ± 0.8** | **1.8 ± 0.6** | **0.001** |  | **2.1 ± 0.8** | **1.8 ± 0.6** | **<0.001** |
| Causal belief: genetics/heredity^†^ (1-5) | 4.1 ± 0.7 | 4.0 ± 0.9 | 0.777 |  | 4.2 ± 0.9 | 4.1 ± 0.8 | 0.240 |  | 4.2 ± 0.8 | 4.1 ± 0.8 | 0.290 |
| Causal belief: lifestyle^†^ (1-5) | 3.3 ± 1.3 | 3.5 ± 1.2 | 0.491 |  | 3.7 ± 1.1 | 3.4 ± 1.1 | 0.132 |  | 3.5 ± 1.2 | 3.4 ± 1.1 | 0.426 |
|  |  |  |  |  |  |  |  |  |  |  |  |
| *Binary Measures* | *%* | *%* | *p* |  | *%* | *%* | *p* |  | *%* | *%* | *p* |
| Interest in genetic risk assessment | **80%** | **96%** | **<0.001** |  | **90%** | **98%** | **<0.001** |  | **86%** | **98%** | **<0.001** |
| Expectation of reassurance^†^ | 12% | 17% | 0.503 |  | 11% | 21% | 0.080 |  | 11% | 20% | 0.061 |
| Expectation of aided decision making^†^ | 15% | 18% | 0.791 |  | 18% | 17% | 0.875 |  | 17% | 17% | 0.988 |

* Assessed during the telephone interview (196 actively recruited participants, 484 self-referred participants, 680 total)

† Assessed in the baseline written questionnaire (163 actively recruited participants, 444 self-referred participants, 607 total)
