# Supplementary material for: Associations between self-referral and health behavior responses to genetic risk information
Source: Genome Med. 2015 Jan 31;7(1):10. doi: 10.1186/s13073-014-0124-0 (PMC4311425; doi:10.1186/s13073-014-0124-0)
Supplement: Additional file 5: — Additional REVEAL Study Group members. [file 13073_2014_124_MOESM5_ESM.docx]

## Additional REVEAL Study Group members

Additional members of the REVEAL Study group are as follows: D. Bhatt, Brigham and Women’s Hospital, Boston; D. Blacker, Mass General Hospital/Harvard Medical School an Harvard School of Public Health, Boston; M. Butson, Case Western Reserve University, Cleveland; C. Chen, Boston University School of Public Health, Boston; R. Cook-Deegan, Duke University, Durham; E. Cox, Weill Cornell Medical College, New York; L. Cupples, Boston University School of Public Health, Boston; J. Davis, Weill Cornell Medical College, New York; L. Farrer, Boston University School of Medicine and Boston University School of Public Health, Boston; G. Fasaye, Inova Cancer Center, Fairfax; P. Griffith, Morehouse School of Medicine, Atlanta; K. Harkins, Perelman School of Medicine, Philadelphia; S. Hiraki, GeneDX, Gaithersburg; M. Johnson, Howard University, Washington, DC; S. Johnson, Howard University, Washington, DC; E. Juengst, University of North Carolina School of Medicine, Chapel Hill; J. Karlawish, Perelman School of Medicine, Philadelphia; D. Lautenbach, Brigham and Women’s Hospital, Boston; L. Le, University of Michigan School of Public Health, Ann Arbor; E. Levison, Division of Genetics, New York Presbyterian Hospital; E, McCarty Wood, Perelman School of Medicine, Philadelphia; T. Obisesan, Howard University, Washington, DC; S. Post, Stony Brook University, Stony Brook; K. Quaid, Indiana University School of Medicine, Indianapolis; L. Ravdin, Weill Cornell Medical College, New York; N. Relkin, Weill Medical College of Cornell University; D. Roter, Johns Hopkins Bloomberg School of Public Health, Baltimore; C. Royal, Duke University, Durham; R. Stern, Boston University School of Medicine, Boston; A. Sadovnick, University of British Columbia, Vancouver; S. Sami, Case Western Reserve University, Cleveland; P. Sankar, Perelman School of Medicine, Philadelphia; E. Topol, Scripps Research Institute, La Jolla; W. Uhlmann, University of Michigan, Ann Arbor; L. Waterston, Maine Medical Center, Portland; P. Whitehouse, Case Western Reserve University, Cleveland; L. Wright, Medical College of Georgia, Athens.
